# A Practical Treatise on Fractures and Dislocations

**Published:** 1891-07

**Authors:** 


A Practical Treatise on Fractures and Dislocations. By Frank Hastings Hamilton, A. B., A. M., M. D., LL. D., late Professor of Surgery in Bellevue Hospital Medical College, and Surgeon to Bellevue Hospital, New York; Consulting Surgeon to Hospital for Ruptured and Crippled, to Elizabeth's Hospital, etc.; author of a Treatise on Military Surgery and Hygiene; a Treatise on the Principles and Practice of Surgery, etc. Eighth edition, revised and edited by Stephen Smith, A. M., M. D., Professor of Clinical Surgery in the University of the City of New York, and Surgeon to Bellevue and St. Vincent's Hospitals, New York. Illustrated with 507 wood-cuts. Pp. xvi.—849. Philadelphia: Lea Brothers & Co. 1891. Price, cloth, $5.50; sheep, $6.50.

This classical treatise has more than ordinary interest for the medical profession of Buffalo, for in the old tenement building upon the corner of Virginia street and Pearl place, where once was the Sisters of Charity Hospital, the author gained the experience and recorded the cases which form no inconsiderable part of this admirable work.

Dr, Smith has brought the book quite up to modern standards, a task which he is peculiarly ably fitted to perform, inasmuch as he was for so long a time intimately associated with the author, first as pupil and then as colleague, and had an important share in the collection and arrangement of the materials out of which this book grew. Dr. Smith has, furthermore, made the text more compact, eliminated matter not bearing upon the case, and utilized his own large clinical experience in many instances. When a book has reached an eighth edition there remains little to say concerning it that has not already been said. The fact that it has reached an eighth edition, and has been translated into many languages, alone attests its great worth. With Dr. Smith to supervise it in the future, it cannot fail to be what it has always been, a sterling guide to the practitioner and student.

The publishers have done their part in their usual excellent way. One hundred and five new illustrations have been added.

